# Editorial: New methods for old dilemmas

**DOI:** 10.3389/fped.2023.1289472

**Published:** 2023-09-29

**Authors:** Chad B. Crigger, John P. Gearhart

**Affiliations:** Department of Urology, Division of Pediatric Urology, Johns Hopkins Hospital, Baltimore, MD, United States

**Keywords:** outcomes, management updates, methodology, pediatric surgery, quality

**Editorial on the Research Topic** Methods in pediatric urology 2022

Pediatric urology and surgery require a greater appreciation for delicate tissue coupled with the creativity to address significant challenges in petite patients, a balance not typically seen in adult patients. The need to constantly improve techniques and usher in newer iterations drives much of the methodology seen in pediatric surgery with the paramount goal of improving (or at least maintaining outcomes) while limiting, as much as possible, any associated morbidity—a lofty task indeed.

This research topic, *Methods in Pediatric Urology*, represents this goal—applying techniques in the pediatric realm that provide updates and slight refinements on existing methodologies. All of the articles that follow in this topic share the unifying theme of providing further contemporary insight on historically challenging topics. For instance, cryptorchid testicular torsion, encountered by very few in practice, may actually be present in up to 15% of cases according to the literature. Chen et al. reaffirmed this notion, citing a 10.2% incidence in the number of testicular torsions during their study period. With proper diagnostic tools, including deft ultrasonography and mean platelet volume, along with a low diagnostic threshold for often obscure symptomatology, the authors offer additional clarification of a diagnostic dilemma to better equip practicing urologists.

Continuing in this theme in the management of upper kidney preservation for complete duplication, Chu et al. elegantly provide further evidence for laparoscopic ureteral end-to-side anastomosis (UU) compared to minimally invasive ureteral reimplantation (UC). The authors found overwhelming statistically better outcomes with UU in nearly all regards, including operative time, drainage time, degree of hydronephrosis and length of stay. This contribution also excels in providing tips-and-tricks embedded within the paper, a delight not typically seen in a methodology manuscript.

In pediatric trauma, much of what is actually practiced is distilled from adult practice—a challenge when considering the anatomic differences between children and adults. Zhu et al. provide impressive outcomes for transperineal anastomotic urethroplasty for posterior urethral stenosis. In this series, the authors achieved an 85.7% success rate (24/28) with normal postoperative voiding for 26 of 28 patients after two underwent additional surgery with the remaining two still awaiting surgery. Importantly, Zhu et al. provide a technique roadmap including splitting of the corpus cavernous septum or partial resection of the lower aspect of the pubic symphysis, techniques not often employed in the pediatric population. With sensitivity, the authors acknowledge that such an approach does require longer term follow up to evaluate for any eventual sexual dysfunction that may arise.

As a bonus, even laparoscopic resection of choledochal cysts and Roux-en-Y reconstruction was included in this pediatric surgery topic. With 33 cases performed, without open conversion, and eventual success in all 33 after conservative management in 4 for pancreatitis (2) and biliary fistula (2) Bian et al. provide further evidence for minimally invasive management of this complex pathology.

This research topic is the result of the efforts of many, including the topic editors, coordinators, and of course, the numerous reviewers who kindly tolerated electronic reminders and met deadlines with aplomb. We hope this research topic provides an interesting update to the existing surgical methodology discussed and will remain a reference in the future.

